# A comparative study on the mechanical, physical and morphological properties of cement-micro/nanoFe_3_O_4_ composite

**DOI:** 10.1038/s41598-020-59846-y

**Published:** 2020-02-18

**Authors:** Siamak Imanian Ghazanlou, Maisam Jalaly, Sadegh Sadeghzadeh, Asghar Habibnejad Korayem

**Affiliations:** 1 Nanotechnology Department, School of Advanced Technologies, Iran University of Science & Technology (IUST), Narmak, Tehran 16846-13114 Iran; 2 School of Civil Engineering, Iran University of Science & Technology (IUST), Narmak, Tehran 16846-13114 Iran

**Keywords:** Civil engineering, Nanoparticles

## Abstract

In this study, fabrication of a composite containing the ordinary Portland cement (OPC) and magnetite (Fe_3_O_4_) micro/nanoparticles is reported. In the first stage, the cement paste samples with a fixed 0.2 wt.% Fe_3_O_4_ additive in four different particle sizes (20–40 nm, 80–100 nm, 250–300 nm, and 1–2 µm) were prepared to check the effect of magnetite size. Magnetite was found to play an effective role in reinforcing cement matrix. The results showed that the cement paste reinforced by magnetite nanoparticles of 20–40 nm size range had the highest compressive, flexural, and tensile strengths compared to those of the other samples reinforced by larger particles. In the second stage, various amounts of the Fe_3_O_4_ nanoparticles of 20–40 nm size range were added to the cement to evaluate the influence of magnetite amount and find the optimized reinforcement amount. It was revealed that adding 0.25 wt.% Fe_3_O_4_ nanoparticles of 20–40 nm size range, as the optimal specimen, increased the compressive strength, flexural strength and tensile splitting strength by 23–32, 17–25, and 15–19%, respectively, and decreased the electrical resistance by 19–31%.

## Introduction

In recent years, attention to the environmental aspects of materials has resulted in major changes in the composition of Portland cement to meet the growing needs in the construction sector better^1^. One of the most beneficial compositional reforms is addition of reinforcing fillers into the chemical composition of cement^1^. Among these additives, nanoparticles have attracted more attention and they are being used in many areas to produce new materials with new functions because of their unique physical and chemical properties^2^. If nanomaterials are added into the cementitious materials, a composite creates that may have enhanced properties^2^. However, addition of nanoparticles in the cement may suffer from their non-uniform distribution within the matrix, which can reduce the mechanical properties of the block. The main bottleneck of using the concrete is the brittle nature of its fracture that can be attributed to its low tensile strength, low strain capacity, and low resistance to cracking. Depending on the cement/aggregate ratio and the amounts of cement and water in the mixture, the tensile strength of concrete ranges from 2 to 8 MPa^3^.

Many research studies have assessed the effects of nanoparticles on the various properties of the cement and concrete over the last few years. SiO_2_, CaCO_3_, TiO_2_, ZrO_2_, Al_2_O_3_, and Fe_2_O_3_ nanoparticles as well as carbon nanomaterials such as graphene oxide and carbon nanotubes are mostly used nanostructures in the cementitious matrices^4–10^.

Iron oxide has several chemical forms among which FeO, Fe_2_O_3_, and Fe_3_O_4_ are the most common phases. It has been previously reported that the addition of Fe_2_O_3_ (hematite) nanoparticles can improve the mechanical properties (e.g. 8% increase in compressive strength by adding 3% Fe_2_O_3_ in 90 hydration days) and durability of the concrete, and concurrently reduce its permeability (e.g. 44% decrease)^11,12^. Previous studies have shown that adding Fe_2_O_3_ nanoparticles, in addition to increasing the compressive strength, increases flexural and tensile strengths^13–15^. Nano-hematite was also shown to have a better self-monitoring capability than nano-SiO_2_ additive^16^. Furthermore, Fe_3_O_4_ (magnetite) nanoparticles were fruitfully added into the concrete for shielding of microwave radiation^17^. The concrete containing 1.5 wt.% Fe_3_O_4_ nanoparticles indicated the lowest water absorption and highest tensile strength compared to the concrete reinforced by the same amount of ZrO_2_, Al_2_O_3_ and TiO_2_ nanoparticles^18^. Moreover, Amin *et al*.^19^ showed that the addition of low percentages of nano-magnetite to the cement paste improved compressive strength during all ages of the hydration process. Fe_3_O_4_ nanoparticles were observed to act as a filler for cement paste, and refine pore structures, reduce total porosity, and increase the density of the cement composites^20^.

Although the introduction of magnetite particles into the cementitious materials has already been studied in a few works as mentioned above, there is still some uncertainty regarding the mechanism by which and the extent to which the magnetite additive can affect the cement. This ambiguity needs to be clarified. The current work is a systematic investigation to respond this need. The purpose of the present study is to evaluate the effects of size and amount of Fe_3_O_4_ filler in the different hydration ages comprehensively on various mechanical, physical, and morphological properties of the cement paste, which has not been reported so far to the best of our knowledge. The outcomes of this research could pave the way to fabricate high performance concrete and achieve a sustainable concrete structure.

## Materials and Methods

Ordinary Portland cement (OPC) was used as received and conformed the requirements of ASTM C150 standard. The chemical composition of OPC analyzed by X-ray fluorescence (XRF) is shown in Table S1. Analytical-grade magnetite (Fe_3_O_4_) powders with four different particle sizes of 20–40 nm (F1), 80–100 nm (F2), 250–300 nm (F3), and 1–2 µm (F4) were used without any further treatment. Magnetite powders with purity of >98% were supplied from VCN Materials Co. (I.R. Iran).

The cement paste was prepared using a high-speed shear mixer (TAT-2500). The nano-magnetite powder was first dispersed into the distilled water by an ultrasonic probe for 30 min and the prepared colloid was poured into the mixer bowl containing the cement. Next, the paste was mixed at 2500 rpm for 5 min. The water/cement ratio was 0.5 throughout the study. In the supplementary file, Fig. S1 shows the schematic flow chart for samples preparation. In the first run of experiments, the cement composites containing constant fraction of 0.2 wt.% Fe_3_O_4_ of different particle sizes (F1–F4) were fabricated. In the second run of experiments, the optimum sample of the first run was chosen to evaluate the different fractions of magnetite additive. Table 1 presents the details of the mixture proportions.

**Table 1:** Mixture proportions for different specimens.

	Sample code	Water/cement ratio	Magnetite particle size	Magnetite/cement percentage (wt.%)
First Stage of the work	C	0.5	—	0
	F1	0.5	20–40 nm	0.20
	F2	0.5	80–100 nm	0.20
	F3	0.5	250–300 nm	0.20
	F4	0.5	1–2 µm	0.20
Second Stage of the work	F1–10	0.5	20–40 nm	0.10
	F1–15	0.5	20–40 nm	0.15
	F1–20	0.5	20–40 nm	0.20
	F1–25	0.5	20–40 nm	0.25
	F1–30	0.5	20–40 nm	0.30
	F1–35	0.5	20–40 nm	0.35

Fresh cement paste was poured into the corresponding molds for various tests. The shapes of the molds were as follows: cubes of 15 mm × 15 mm × 15 mm for compressive strength, cubes of 10 mm × 10 mm × 50 mm for flexural strength, cubes of 50 mm × 10 mm × 10 mm for electrical resistivity, cylinders of Φ15mm × 30 mm for tensile strength, cylinders of Φ25mm × 50 mm for porosimetry, and cylinders of Φ50 mm × 50mm for capillary water absorption. The specimens were removed from the molds after 24 h and immersed in a lime-saturated reservoir at 25 °C.

The compressive strength was measured according to the method prescribed by ASTM C109/C109M-11b using a displacement rate of 2 mm/min. In the area after the failure, linear voltage displacement transducers (LVDTs) were used to acquire the stress/strain data, because reading the pressure gauges were unreliable after reaching the peak point due to the sample cracking^21^. Two LVDTs were placed on the left and right sides of the specimen to obtain the average displacement (see Fig. S2). The strain was further measured using a laser extensometer of LX 500. In all stress/strain curves, axial strains were confirmed using these two methods of measurement. A high-speed data acquisition system was used to achieve data including loads and pressures.

The flexural strength was measured using an STB 504 three-point flexural bending machine following the procedure described by ASTM C78/C78M-10. Flexural strength tests were performed on the specimens using a displacement rate of 0.2 mm/min and the maximum load for each specimen was attained over a period of 60–100 s. To measure the tensile strength of specimens, Brazilian tensile test was used in accordance with ASTM C496 using a STB 504 machine with a capacity of 50 kN and loading rate of 2 kN/min.

The total porosities of the samples were measured by a helium pycnometer. The samples cured for different days were dried at 105 °C and subjected to the pycnometry. The measurements of pulse velocity were made by a commercially portable ultrasonic pulse tester with 54 kHz transducers using the direct transmission mode according to the standard ASTM C597–16. Electrical resistivity values of the cement-based samples were measured using the simple two-electrode method. Copper plates with a width of 5 mm were attached to the specimens as the contact electrodes. A concrete resistance gauge (RCON, Giatec) was employed to record the impedance between two copper electrodes. Figure S3 displays a schematic image from the setup used for measuring the resistivity. The AC current was applied between electrodes, so that the drive time of each frequency was about 5 s. The bulk resistivity (Ω.m) can be calculated using the following equation^7^:

1 $$\rho =R\frac{A}{L}$$

where *R* (Ω) is the electrical resistance, *A* is the cross-sectional area of the sample (10 mm × 10 mm) and *L* is the distance between the two contacts (50 mm).

Capillary water absorptions of the samples were assessed in accordance with ASTM C1585–13. The specimens were removed from their molds after their corresponding ages and dried at 100 °C for 24 h. The specimens were then partially immersed in the water for different durations, and the other surfaces were covered by waterproof sealants. The capillary sorptivity (mm/s^0.5^) was calculated using the following equation:

2 $$S=\frac{{V}_{w}}{A\sqrt{t}}$$

where *V*_*w*_ is the volume of the water absorbed (mm^3^), *A* is the surface area (mm^2^), and *t* is the exposure time (s). Initial and secondary sorptivities were measured during the first 6 hours and 1–7 days in water absorption test, respectively^22^. It is worth noting that each test result is an average of the same results achieved from five specimens.

A Panalytical X’Pert diffractometer (45 kV, 40 mA) with Cu Ka radiation (k = 0.15406 nm) was used for the X-ray diffraction (XRD) analysis. A JEOL 7001 F FEG scanning electron microscopy (SEM) equipped with an electron gun with a cold emission was used to check the morphologies of the different Fe_3_O_4_ powders (Fig. S4) as well as the fracture surfaces of the cementitious blocks. The fracture surface was sputtered with a thin layer of Pt/Pd prior to SEM imaging.

## Results and Discussion

### The effect of magnetite particles size

As pointed out above, in the first stage of the work, different magnetite sources with different particle sizes were added to the cement paste to evaluate the effect of reinforcement sizes on the mechanical and physical features of the cement and to find the optimum sample.

#### Compressive strength

**Figure 1:**
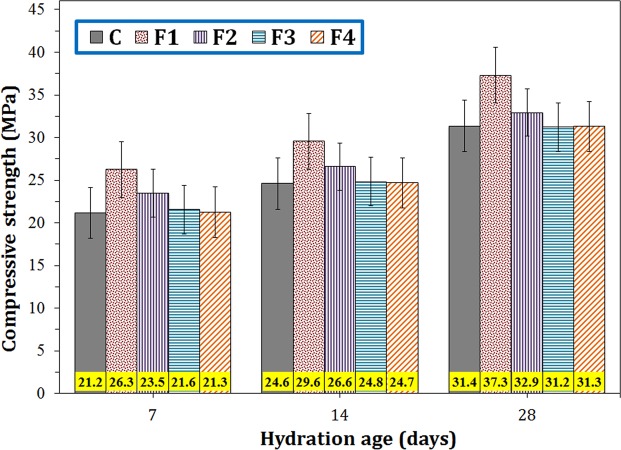
Compressive strengths of the cement paste containing 0.2 wt.% Fe_3_O_4_ with different particle sizes nanoparticles at different hydration ages.

Figure 1 shows the compressive strengths of the cement paste containing constant 0.2 wt.% magnetite additives with four different particle sizes (samples F1–F4) within different hydration ages. As shown in this figure, almost all magnetite additives increased the compressive strength of the neat cement paste (sample C), but the sample containing Fe_3_O_4_ particles of 1–2 µm (sample F4) had an insignificant effect on the compressive strength. As Fig. 1 shows, Fe_3_O_4_ additives resulted in an increase in compressive strengths at all ages of hydration in the following order: F1 > F2 > F3 > F4 > C. The highest compressive strength was achieved in case of sample F1. By adding 0.2 wt.% Fe_3_O_4_ nanoparticles of 20–40 nm size range (sample F1), the compressive strength increased by ~24, 20, and 19% for the hydration ages of 7, 14 and 28 days, respectively. Regarding the error bars of the data, it is clear that the fruitful effect of the nanoscale filler is not within the technical error ranges.

**Figure 2:**
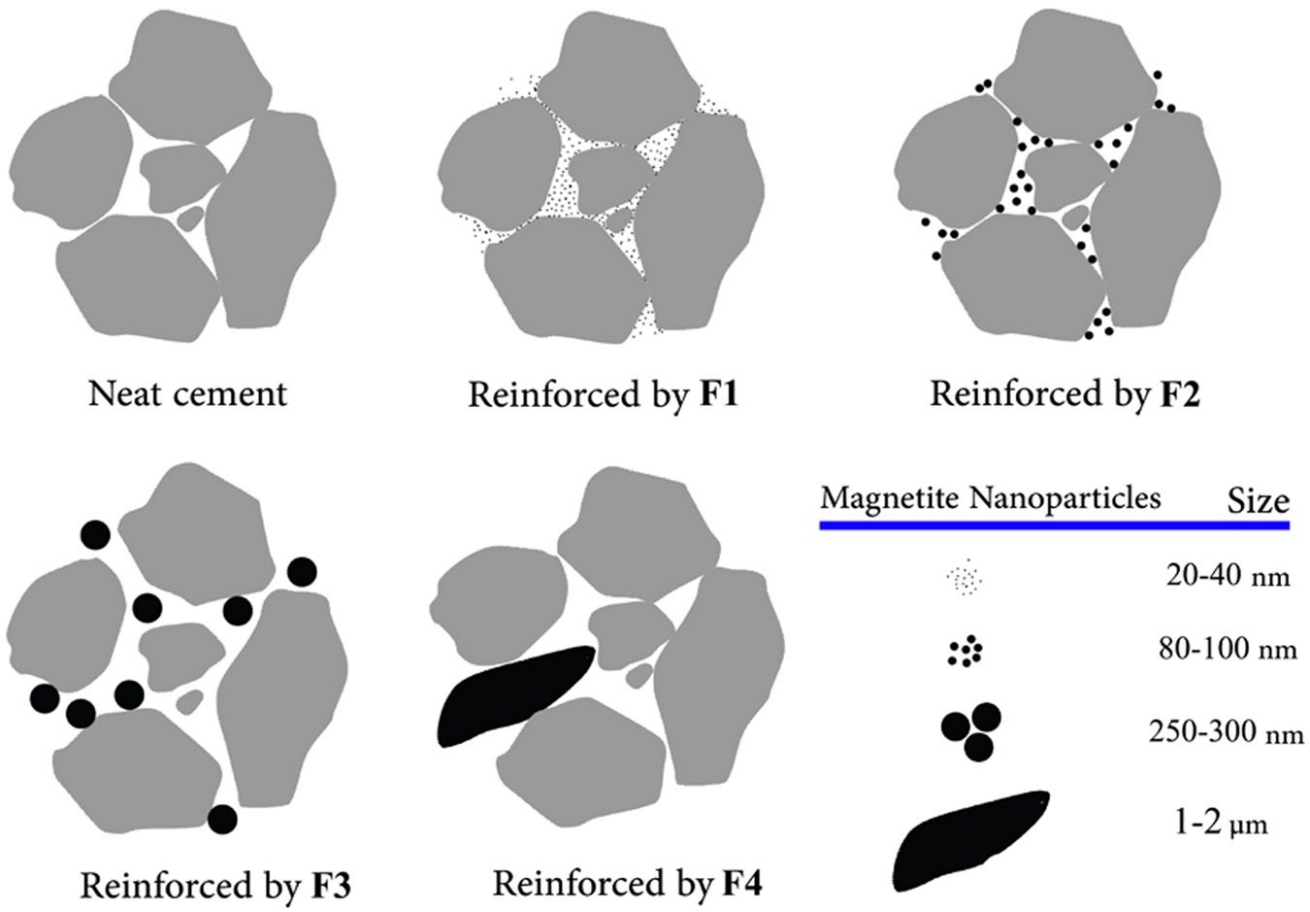
Schematic dispersion of differently sized magnetite additives in the cement pore structure.

The samples containing nanoparticles (F1 and F2) showed greater improvements in compressive strengths, implying that particulate additives with nanoscale size have a more remarkable reinforcing effect than micrometer/submicrometer particles. It can be observed that both F1 and F2 samples exhibited further increases in compressive strengths within less hydration ages that can be attributed to the modification and/or acceleration of hydration reaction by Fe_3_O_4_ nanoparticles^19^. The roles of F3 and F4 additives in changing the compressive strength of the cement were negligible most likely due to their large magnetite particle size and their low ability to reside in the pores, thereby reduction of their filling capability and hydration acceleration. In fact, small nanoparticles can easily fill the pores of the cement and provide a better dispersed composite, while large Fe_3_O_4_ additives are not able to locate in small cavities, pores or cracks, providing an undesired dispersion in the composite (see Fig. 2). Furthermore, the presence of the nanoparticles within cement matrix increases the nucleation sites, leading to the increased amount of the CH and C-S-H phases which will be shown in the XRD section. This may be the reason for acceleration of the hydration process.

#### Flexural and tensile strengths

**Figure 3:**
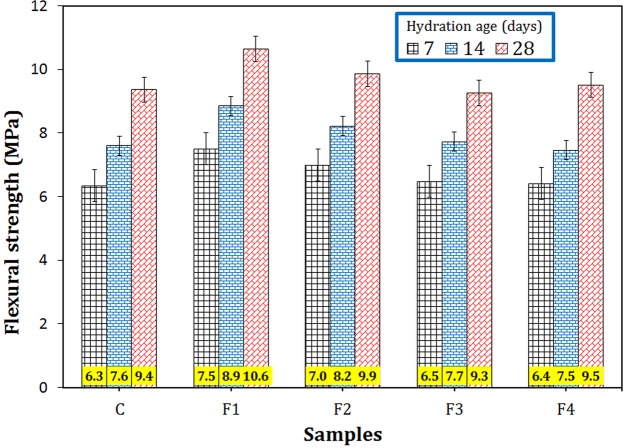
Flexural strength of the cement paste containing 0.2 wt.% Fe_3_O_4_ with different particle sizes nanoparticles at different hydration ages.

The flexural strengths of the different samples within the ages of 7, 14, and 28 days are depicted in Fig. 3. The splitting tensile strength values of the cement paste containing different magnetite additives are shown in Fig. S5. Almost all four types of Fe_3_O_4_ particles increased the tensile strength of the cement paste, but the samples reinforced by Fe_3_O_4_ nanoparticles had the highest flexural and tensile strengths among other samples. By adding 0.2 wt.% Fe_3_O_4_ nanoparticles of 20–40 nm size range (sample F1), the flexural and tensile strengths increased by 18–13 and 12–8%, respectively, for the hydration ages of 7–28 days, which is due to the improvement of the filling capability.

#### Stress–strain diagrams

**Figure 4:**
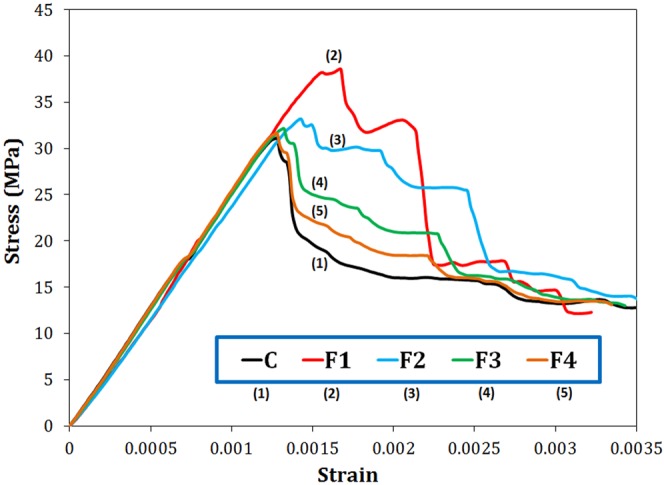
Compressive stress-strain curves of the cement paste containing 0.2 wt.% Fe_3_O_4_ with different particle sizes cured for 28 days.

The compressive stress-strain curves for the neat cement sample (C) and samples reinforced by magnetite particles of different sizes (F1–F4) cured for 28 days are shown in the Fig. 4. The elastic moduli (*E*) of various samples were calculated from the slope of the linear part of the corresponding *σ*–*ε* curves. The elastic modulus was obtained as about 25 GPa for all samples. Magnetite additives had no notable influences on the *E*s of different samples, since the elastic modulus of a composite is mainly affected by the volume fraction of its reinforcing filler^23^, and a fixed, small fraction of magnetite additive was used in the current work.

Figure 4 exhibits that the highest strain values belong to F1 followed by F2, suggesting that smaller magnetite nanoparticles can further increase the ductility of the cement. In the post-peak area, the curves exhibited descending tails, which is an indicator for brittle materials. However, the samples F1 and F2 showed a more delayed fracture, representing tougher nature of these samples. Toughness of the samples was calculated by integrating the compressive stress-strain curve. The values of 0.020, 0.034, 0.024, 0.020, and 0.020 J/m^3^ were achieved for toughness of C, F1, F2, F3, and F4 samples, respectively. It is obvious that the smallest Fe_3_O_4_ nanoparticles (F1) improved the cement paste toughness by 70%, and they were the most useful additives. The load-displacement curves resulted from tensile test for these simples hydrated for 28 days are also shown in Fig. S6. It can be seen that the highest ultimate displacement was obtained in the cement loaded with the smallest magnetite nanoparticles, F1, implying higher load endurance at higher stresses. In addition, F1 shows the largest area under the curves. In fact, neat cement paste is highly brittle due to its high porosity, large pores and, consequently, the rapid growth of cracks^24^. In the reinforced composites, magnetite nanoparticles (especially F1 sample) can easily locate between cement grains and act ahead of crack tip as a reinforcing filler, preventing the propagation of microcracks by elastic crack pinning^25^.

#### Ultrasonic pulse velocity

Air voids were determined by measuring ultrasonic pulse velocity. The wave pulses are sent to the cementitious samples and the time taken by the pulse to get through the samples is measured. Higher velocities imply better quality and more continuity of the structure; whereas lower velocities indicate that the samples may contain voids and cracks. Figure S7 displays the velocities measured for various samples after 3, 7, and 28 days of hydration. In general, the pulse velocities in different samples are close to each other, indicating little entrapment of large voids during sample preparation. Nonetheless, the sample F1 exhibits the highest pulse velocity, suggesting that small nanoparticles can further fill the air voids and avoid microcracks from further opening.

#### Porosity

**Figure 5:**
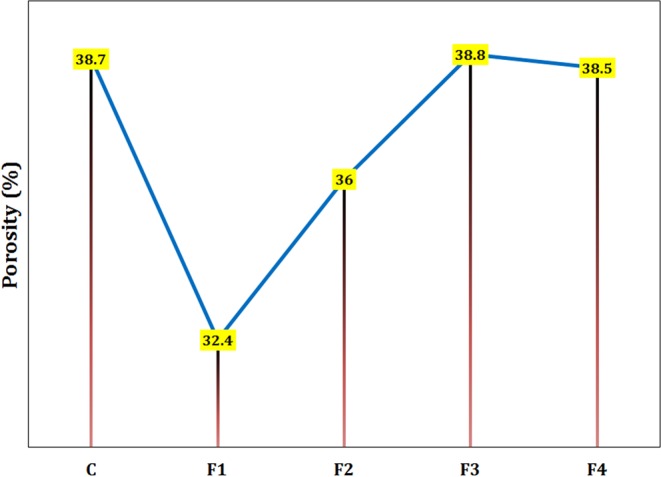
The porosity for various samples containing 0.2 wt.% Fe_3_O_4_ with different particle sizes hydrated for 28 days.

The porosities of the neat cement paste and cement-based composites reinforced by 0.2 wt.% magnetite of different sizes were measured by the helium pycnometry. As shown in Fig. 5, the sample F1 showed the lowest porosity level (32.4%) which is 16.3% less than the porosity of the control sample. There is no significant change in porosity for the samples F3 and F4 most likely due to the large sizes of their additives, which do not affect the filling capacity. The porosity reduction in samples with smaller nanoparticles may be assigned to their better filling capacity and an increase in hydration products due to the enhanced hydration acceleration^22^.

#### Electrical resistivity

**Figure 6:**
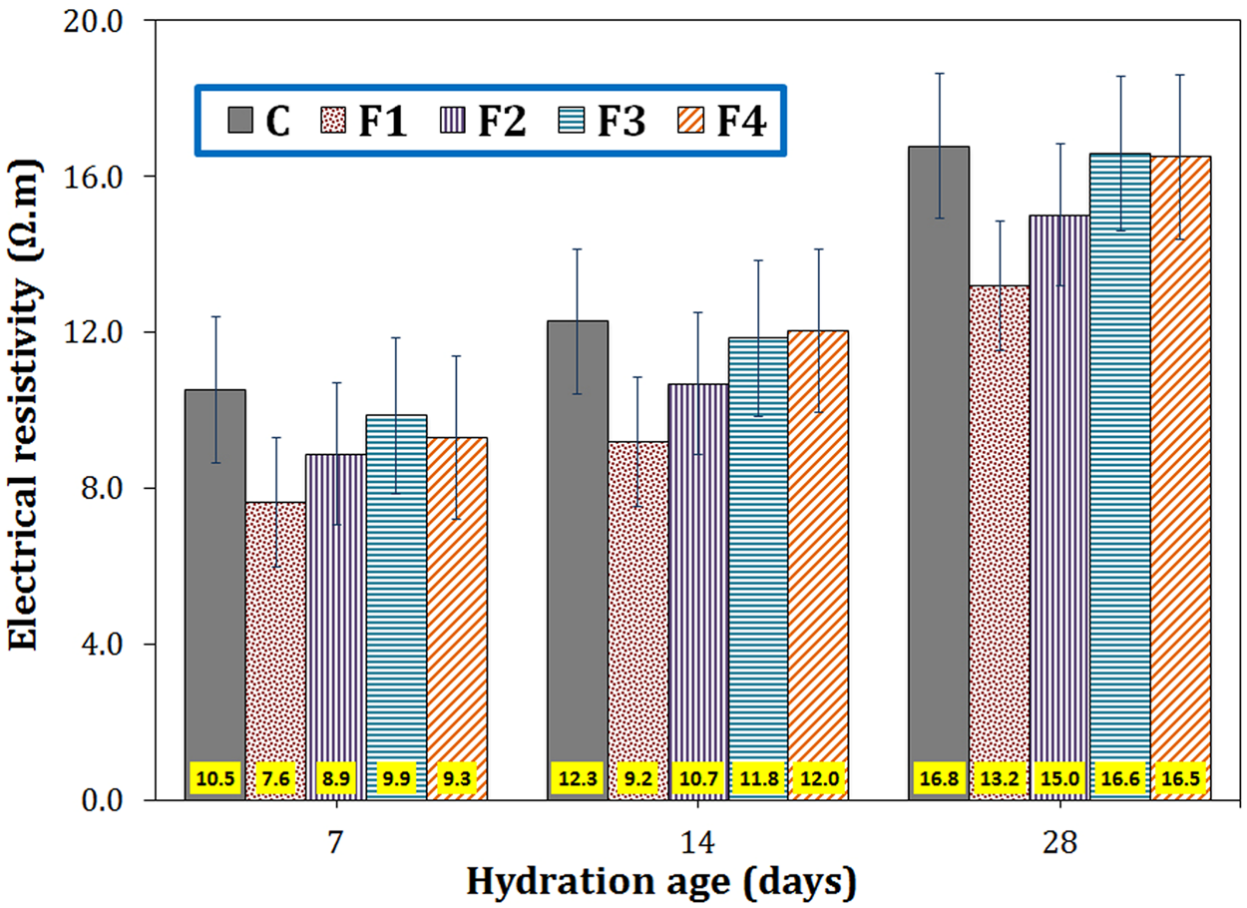
The electrical resistivity of the cement paste containing 0.2 wt.% Fe_3_O_4_ with different particle sizes at 7, 14 and 28 days of hydration.

The effect of the magnetite particle size on the electrical resistivity of the cementitious specimens is shown in Fig. 6. As shown clearly, F1 nanoparticles had the greatest impact on reducing the electrical resistivity (e.g. 27, 25, and 27% drop at 7, 14, and 28 days, respectively). At all ages, adding each type of magnetite particles led to a decrease in resistivity of the cement, which may be attributed to the semiconductor nature of Fe_3_O_4_ particles^26,27^.

#### Water sorptivity

**Figure 7:**
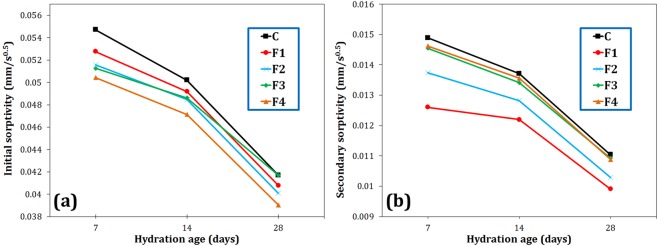
Initial (**a**) and secondary (**b**) sorptivities of the cement paste containing 0.2 wt.% Fe_3_O_4_ with different particle sizes at 7, 14 and 28 days of hydration.

Figure 7 shows the initial and secondary capillary water absorption values of the various samples. The water absorption of the cement was observed to decrease by adding all types of additives that has been also reported already^18,28^. The maximum reduction in initial water absorption was associated with F4 sample at the age of 7 days (7% drop compared to C sample). As the age of the cement increased, the effect of magnetite particles on reducing initial water absorption decreased, because the effect of additives on the hydration acceleration diminished. As shown in Fig. 7(a), the larger the size of the reinforcing filler, the greater the impact on the initial water sorptivity. According to Li *et al*.^29^, the size of the capillary pores can be divided into three groups: (1) large capillaries or macropores (50–10,000 nm), (2) medium capillaries or large mesopores (10–50 nm), and (3) small capillaries or small mesopores (2.5–10 nm). Initial water sorptivity is considered to be mostly affected by macropores, while secondary sorptivity is influenced by small pores^21,29^. In the current study, F4 additive with micrometer-sized particles could fill macropores, more efficiently decreasing initial water absorption. However for the secondary sorptivity, Fig. 7(b) shows that F1 magnetite nanoparticles had the greatest effect on reducing water absorption, since its particle size is within the range corresponding to the large mesopores. F1 decreased the secondary sorptivity of the cement from 0.0148 to 0.0126 mm/s^0.5^ (15% reduction) at 7 days of hydration.

### The effect of the amount of magnetite nanoparticles

According to the results presented above, the sample containing Fe_3_O_4_ nanoparticles of 20–40 nm size range (F1) showed the best results in almost all tests compared to other samples loaded with other magnetite additives. Therefore, in the second stage, the amount of F1 additive was changed to seek the optimum quantity of the magnetite nanoparticles with respect to different mechanical and physical properties. The different samples containing 0.15–0.35 wt.% F1 magnetite nanoparticles were prepared according to Table 1.

#### Compressive strength

**Figure 8:**
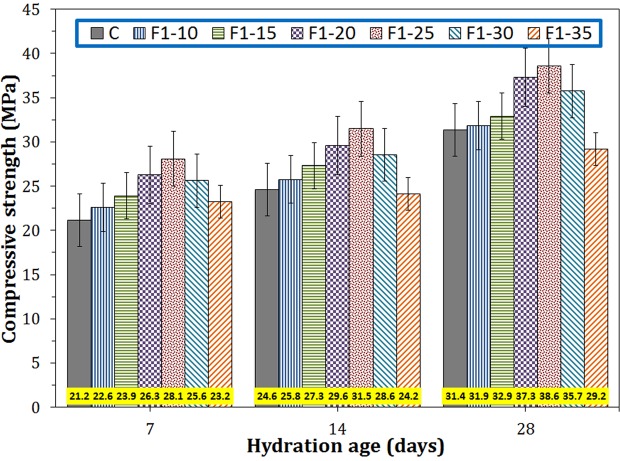
The compressive strengths of the cementitious samples containing different amounts of F1 nano-Fe_3_O_4_ at 7, 14 and 28 days of hydration.

Figure 8 shows the compressive strengths of the cementitious samples containing different amount of Fe_3_O_4_ nanoparticles of 20–40 nm size range at ages of 7, 14, and 28 days. It can be seen that the compressive strength increased initially by increasing nano-magnetite amount up to 0.25 wt.%, and after the maximum point, it decreased with further increase in additive content. Initial rise in the compressive strength is due to the increase of filling capacity of additive and increase of the cement hydration rate. The decrease of the compressive strength after the peak point may be attributed to the agglomeration of magnetite nanoparticles in cement paste in large quantities. The highest compressive strength at all ages was obtained for F1–25 sample which contains 0.25 wt.% of nano-Fe_3_O_4_ (20–40 nm in diameter). The sample F1–25 presented 32, 28, and 23% increase in compressive strength compared to those of the control sample (C) for 7, 14, and 28 days of hydration, respectively. Shakeri and Razzaghi^18^ added 1.5 wt.% nano-Fe_3_O_4_ to the cement and reported an approximately 29% increase in their compressive strength after 28 days of hydration. A 22% increase in the compressive strength of the Portland cement was obtained by Amin *et al*.^19^ by adding 0.3 wt.% Fe_3_O_4_ nanoparticles of 4–7 nm. Sikora *et al*.^20^ also increased the compressive strength of the cement by 21% through loading with 3 wt.% nano-Fe_3_O_4_.

#### Flexural and tensile strengths

**Figure 9:**
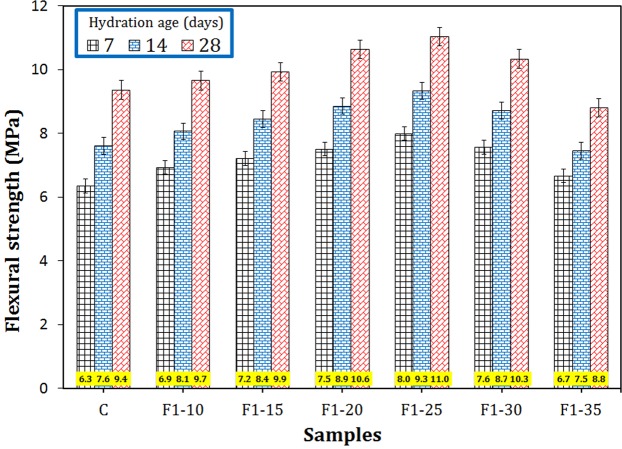
The flexural strengths of the cementitious samples containing different amounts of F1 nano-Fe_3_O_4_ at 7, 14 and 28 days of hydration.

The flexural strengths of the samples containing different amount of F1 additives are shown in Fig. 9. Splitting tensile strengths of these samples are also shown in Fig. S8. In both tests, the sample F1–25 with 0.25 wt.% magnetite nanoparticles exhibits the highest flexural and tensile strengths at all ages (e.g. 25% and 20% increase in comparison with those of C sample at 7 days of hydration for flexural and tensile strengths, respectively). It is worth noting that both flexural and tensile strengths were reduced after this point, so that the flexural and tensile strengths of F1–35 sample, for example, were even lower than the control sample, most likely due to the poor dispersion of highly agglomerated magnetite nanoparticles. For the sake of comparison, Shakeri and Razzaghi^18^ found an approximately 28% increase in tensile strength of the Portland cement after 28 days of hydration by adding 1.5 wt.% nanoFe_3_O_4_.

#### Stress–strain diagrams

**Figure 10:**
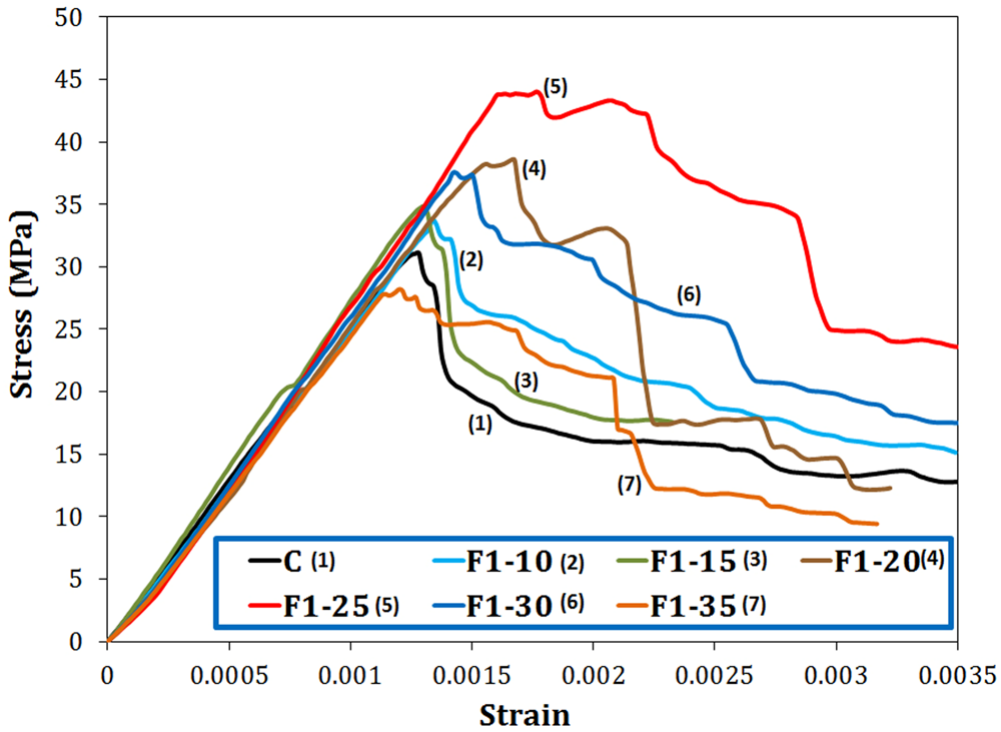
Compressive stress-strain curves of the cement paste containing different amount of F1 nano-Fe_3_O_4_ cured for 28 days.

Figure 10 shows the compressive stress-strain curves for the samples containing different fractions of F1 nanoparticles hydrated for 28 days. The elastic moduli of all specimens were calculated to be about 25 GPa. The extent of nano-magnetite reinforcement seems to be insufficiently great to change the elastic modulus of the cementitious composite.

The yield strain values of the samples are as the following order: F1–25 > F1–20 > F1–30 > F1–15, F1–10 > C > F1–35, indicating that by increasing nano-magnetite additive, ductility of the samples increases initially up to a maximum (0.25% by weight) followed by a decrease. The sample F1–25 exhibited the most delayed failure, implying the highest toughness for this sample. The toughness values were calculated to be 0.020, 0.022, 0.026, 0.034, 0.041, 0.023, and 0.017 J/m^3^ for C, F1–10, F1–15, F1–20, F1–25, F1–30, and F1–35 samples, respectively. The sample F1–25 shows an approximately doubled toughness in comparison with the neat cement paste. The tensile load-displacement diagram for the same samples (Fig. S9) shows that F1–25 sample provides the largest ultimate displacement, the largest area under the curve, and the highest load endurance level.

#### Ultrasonic pulse velocity

Figure S10 shows the pulse velocities measured for the samples containing different nano-magnetite particles at different ages. It is clear that F1–25 additive had a noticeable influence on the wave velocity, and increased it, for instance, by ~11% within 7 days compared to that of the control sample. This is an evidence for reducing the number of small and large cavities in the cement paste by adding magnetite nanoparticles, particularly at early ages, due to the enhancement of the hydration acceleration.

#### Porosity

**Figure 11:**
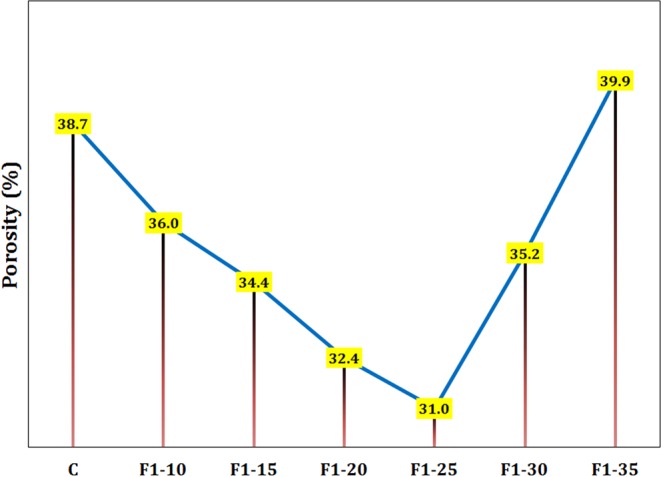
The porosity for various samples containing different amount of F1 nano-Fe_3_O_4_ cured for 28 days.

The effect of different amount of Fe_3_O_4_ nanoparticles on the porosity of cementitious samples is shown in Fig. 11. The sample F1–25 with roughly 20% reduction in porosity compared to that of the neat control sample exhibited the lowest porosity fraction, which is likely due to the high filling ability and increasing hydration products due to the accelerated hydration caused by magnetite nanoparticles. As the amount of nano-Fe_3_O_4_ was greater than 0.25 wt.%, the porosity increased that seems to be because of the agglomeration of the nanoparticles and consequently, reducing their ability to fill the pores.

#### Electrical resistivity

**Figure 12:**
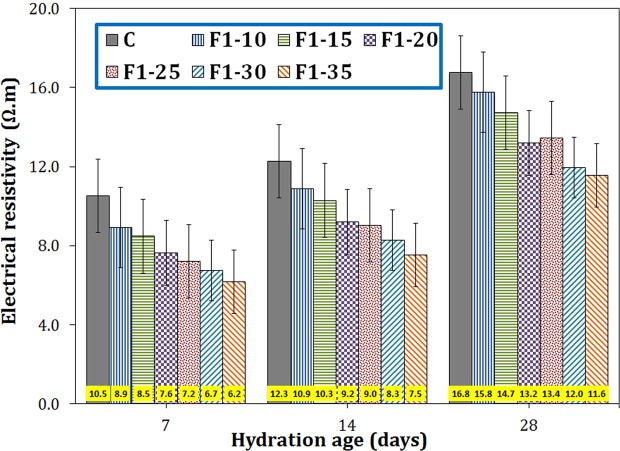
The electrical resistivity of the cement paste containing different amount of F1 nano-Fe_3_O_4_ at 7, 14 and 28 days of hydration.

The electrical resistivity of the cement paste was decreased by increasing the amount of F1 nano-Fe_3_O_4_ at all ages (Fig. 12). The maximum reduction in electrical resistivity occurred within early ages (e.g. 41% fall for F1–35 sample hydrated for 7 days). As the age of the cement increased, the effect of the magnetite nanoparticles on the electrical resistivity of the cement decreased, most likely due to the restriction of the electrical current paths by continuing the cement hydration within later ages.

#### Water sorptivity

**Figure 13:**
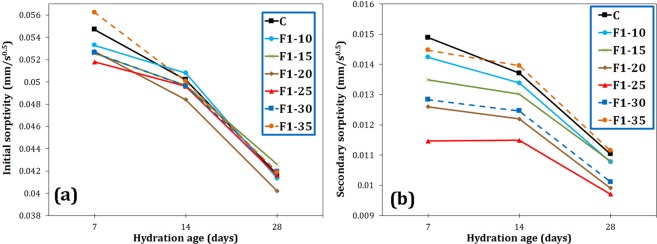
Initial (**a**) and secondary (**b**) sorptivities of the cement paste containing different amount of F1 nano-Fe_3_O_4_ at 7, 14 and 28 days of hydration.

The initial and secondary sorptivity values for different samples with different amount of F1 nanoparticles are shown in Fig. 13. Magnetite nanoparticles of 20–40 nm had no significant effect on the initial water sorptivity (see Fig. 13(a)), because these nanoparticles are not capable of filling macropores. However, these nano-sized additives exhibited a considerable effect on reducing secondary sorptivity (see Fig. 13(b)). The sample F1–25 hydrated for 7 days showed the best performance with an approximately 23% reduction in the secondary sorptivity of the cement paste. Almost all percentages of F1 nanoparticles have improved the secondary sorptivity, although the trend of secondary water sorptivity declined by adding nano-Fe_3_O_4_ more than 0.25 wt.%.

#### XRD analysis

**Figure 14:**
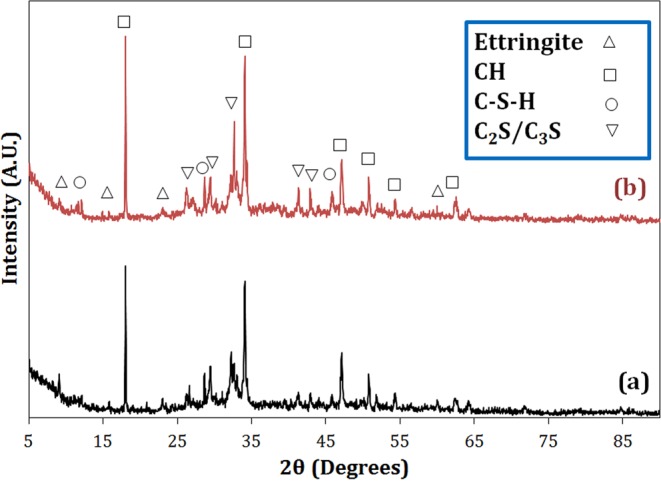
The XRD patterns of the (**a**) C and (**b**) F1–25 samples hydrated for 28 days.

XRD patterns of the neat cement sample and the cement composite loaded with 0.25 wt.% F1 nano-Fe_3_O_4_ cured for 28 days are shown in Fig. 14. The calcium silicate hydrate (C-S-H) phase in the cement behaves like a glue and is responsible for curing the paste to create solid mass, consequently providing a good mechanical strength for the cementitious composites^6^. It can be seen that the intensities of the peaks corresponding to calcium hydroxide (CH, Portlandite) and C-S-H increased by adding magnetite to the cement, which can be attributed to the acceleration of hydration process and the role of nucleation seeding of magnetite nanoparticles, creating a more dense and strong structure^30^. On the other hand, the intensities of the ettringite peaks decreased because the increase of the extent of CH and C-S-H phases inhibited the growth of ettringite crystals.

#### Microstructure study

**Figure 15:**
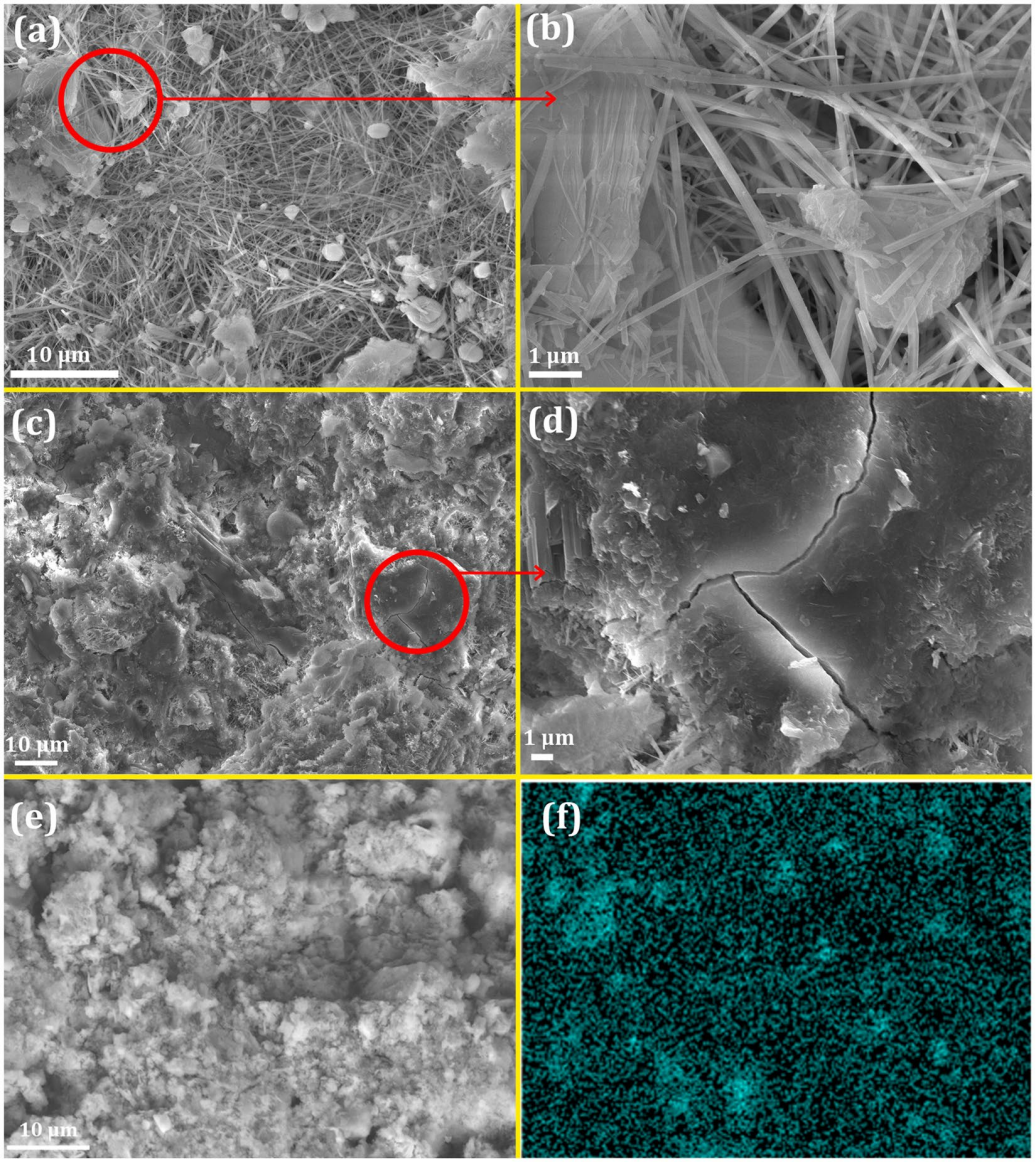
SEM micrographs of the neat cement paste (**a**,**b**) and F1–25 sample (**c**,**d**) hydrated for 28 days. An area (**e**) was selected to show the elemental distribution of Fe (**f**).

The effect of magnetite nanoparticles on the cement hydration and morphology of the hydrated crystals was investigated by the SEM. Figure 15 illustrates the micrographs of the neat cement paste and F1 nano-magnetite reinforced cement hydrated for 28 days. The hydration crystals of the neat cement sample, which usually consist of CH, ettringite, and monosulfonate phases^31^, had randomly stacked needle-like morphology with a diameter of about 100 nm (Fig. 15(a,b)). There are many pores in the hardened cement paste, and cement grains were disintegrated by needle-like ettringite crystals and separated from each other, exerting a very negative effect on the mechanical properties of the cement paste. In contrast, Fig. 15(c,d) indicate that C-S-H gels have been produced in a much denser form in the structure with little cavities, and concurrently, thin needle-like hydration crystals have been removed to a large extent from the structure, confirming the XRD result. A small crack in the cement/Fe_3_O_4_ nanocomposite bifurcated into two separate branches is magnified in Fig. 15(d). In fact, magnetite nanoparticles, as the reinforcing material dispersed in the cement matrix, dissipate the crack propagation energy by deflecting and/or arresting cracks originated from toughening mechanisms including crack pinning, crack bridging, crack branching, etc., resulting in strengthening the brittle cement^25,32,33^. In addition, elemental distribution of Fe in the cement-magnetite nanocomposite is shown in Fig. 15(f) taken from the selected area shown in Fig. 15(e). Uniform distribution of Fe in the microstructure implies a homogeneous dispersion of magnetite nanoparticles in the cementitious matrix.

The improvement of the loosely hardened needle-like structure of the neat cement sample into a compact structure in the cement/nano-Fe_3_O_4_ composite (Fig. 15) was one of the major reasons for enhancing mechanical properties of the cement via the addition of nano-magnetite. The loose structure strongly results in the initiation and propagation of cracks, whereas the compact integrated structure can provide higher resistance against crack propagation. When cracks enter a dense region, they encounter a barrier consisting of tightly integrated hydration crystals, increasing the essential energy for crack growth. Consequently, cracks have to be deflected or branched, hindering crack propagation (Fig. 15(d)).

**Figure 16:**
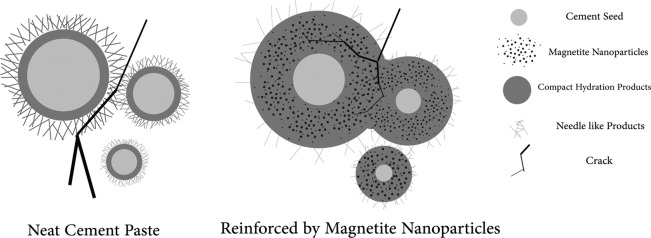
Schematic illustration of crack growth for plain cement and cement/nano-magnetite composite.

As pointed out above, reinforcing nanoparticles can act ahead of crack tips as the crack pinning to toughen the matrix. Residual stresses play a major role in this mechanism. Internal stresses may be generated from different physical properties of matrix (cement) and reinforcing filler (nano-magnetite) of the composite. As a result of this residual stress, the crack being propagated in the matrix deflects toward the nearest nanoparticle due to the stress field around it. Therefore, the nanoparticle barrier can pin the crack tip and impede its growth. Applied stress must be increased to overcome the barrier, implying the strengthening the material. The crack, which leaves the nanoparticle, deflects again to another nearest nanoparticle. This repeated crack deflection hinders the crack propagation by dissipating its energy^25^. Schematic difference in crack propagation between plain cement and cement/nano-magnetite composite is illustrated in Fig. 16. Furthermore, the scheme illustrates better hydration of the cement seeds and reduction of detrimental needle-like phases by adding magnetic nanoparticles.

## Conclusion

In this paper, the composites consisting of cement matrix and magnetite micro/nanoparticles were fabricated. The effects of size and amount of magnetite filler on various mechanical and physical properties of the composites were investigated. The main results can be concluded as follows:

1. Using smaller magnetite particles had more significant effects on improving properties of the cement matrix.
2. In terms of the size effect, the composite sample containing Fe_3_O_4_ particles of 20–40 nm (F1) exhibited 24, 20, and 19% increase in the compressive strength in comparison with the neat cement sample at 7, 14, and 28 days of hydration, respectively, while the effectiveness of other Fe_3_O_4_ fillers of 80–100 nm, 250–300 nm, and 1–2 µm were negligible. Therefore, F1 was the best sample in this stage.
3. In terms of the quantity effect, the optimum amount of magnetite nanoparticles of 20–40 nm was revealed to be 0.25 wt.% (F1–25 sample). Compared to the plain cement, the compressive strength, flexural strength, tensile strength, and ultrasonic pulse velocity of this sample increased by 32, 25, 20, and 11%, respectively, and its porosity, electrical resistivity, and secondary water sorptivity decreased by 20, 41, and 23%, respectively, at early ages.
4. Magnetite nanoparticles gave rise to alter the loose needle-like microstructure of the hardened cement into a compact integrated morphology, which hindered crack propagation by toughening mechanisms such as crack pinning, crack deflection, and crack branching.

## Supplementary information

Supplementary file.
