# Supplementary material for: A comparative study on the mechanical, physical and morphological properties of cement-micro/nanoFe3O4 composite
Source: Sci Rep. 2020 Feb 18;10:2859. doi: 10.1038/s41598-020-59846-y (PMC7028720; doi:10.1038/s41598-020-59846-y)
Supplement: Supplementary file 1 — Supplementary file. [file 41598_2020_59846_MOESM1_ESM.pdf]

Supplementary Information

A comparative study on the mechanical, physical and morphological properties of cement-micro/nanoFe_3O_4 composite

Siamak Imanian Ghazanlou [a], Maisam Jalaly [a,*], Sadegh Sadeghzadeh [a], Asghar Habibnejad Korayem [b]

[a] Nanotechnology Department, School of Advanced Technologies, Iran University of Science & Technology (IUST), Narmak, Tehran 16846-13114, Iran
[b] School of Civil Engineering, Iran University of Science & Technology (IUST), Narmak, Tehran 16846-13114, Iran
*Corresponding Author, Email: maisam_jalaly@iust.ac.ir
Tel: +982173225838, Fax: +982177240380

Table S1. Chemical composition (wt.%) of the OPC used in this work.

Phase	CaO	SiO_2	Al_2O_3	Fe_2O_3	MgO	K_2O	Na_2O	SO_3	LOI
Percentage	63.5	19.7	5.1	3.4	1.2	0.6	0.2	2.7	3.4

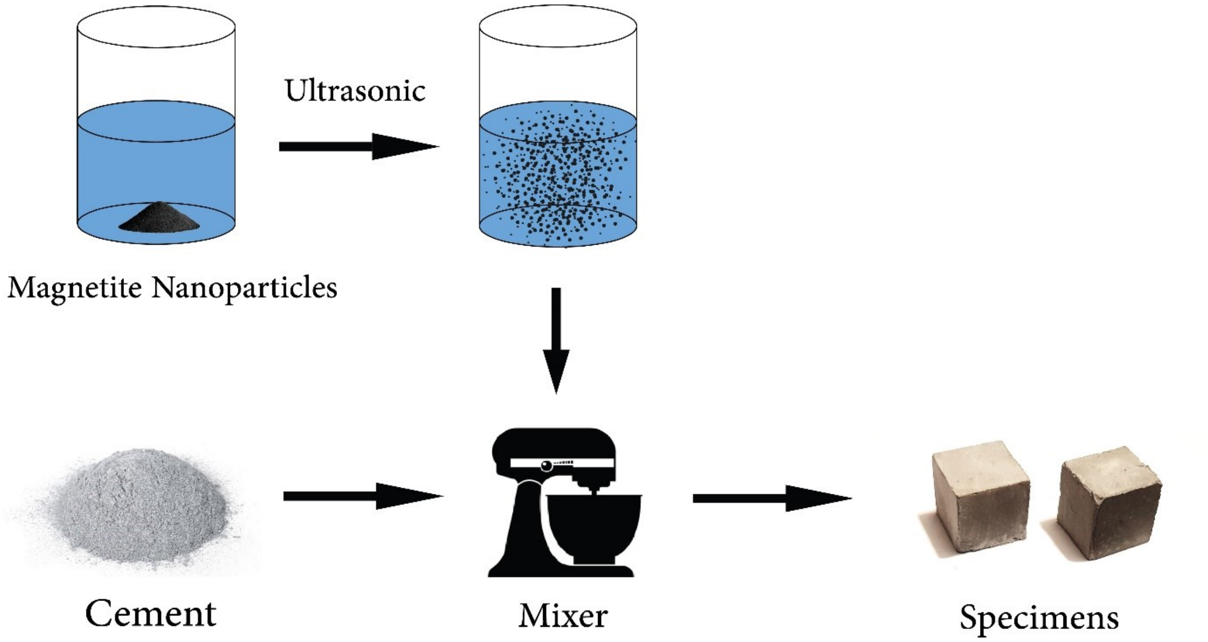

Fig. S1. Schematic flow diagram for preparation of cement paste samples.

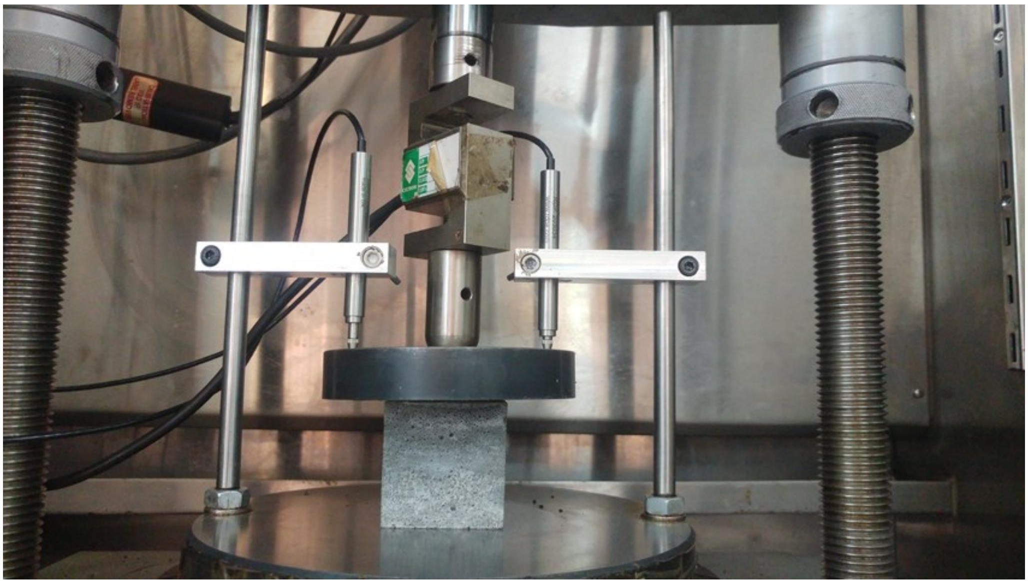

Fig. S2. Digital image from setting LVDTs in compression test.

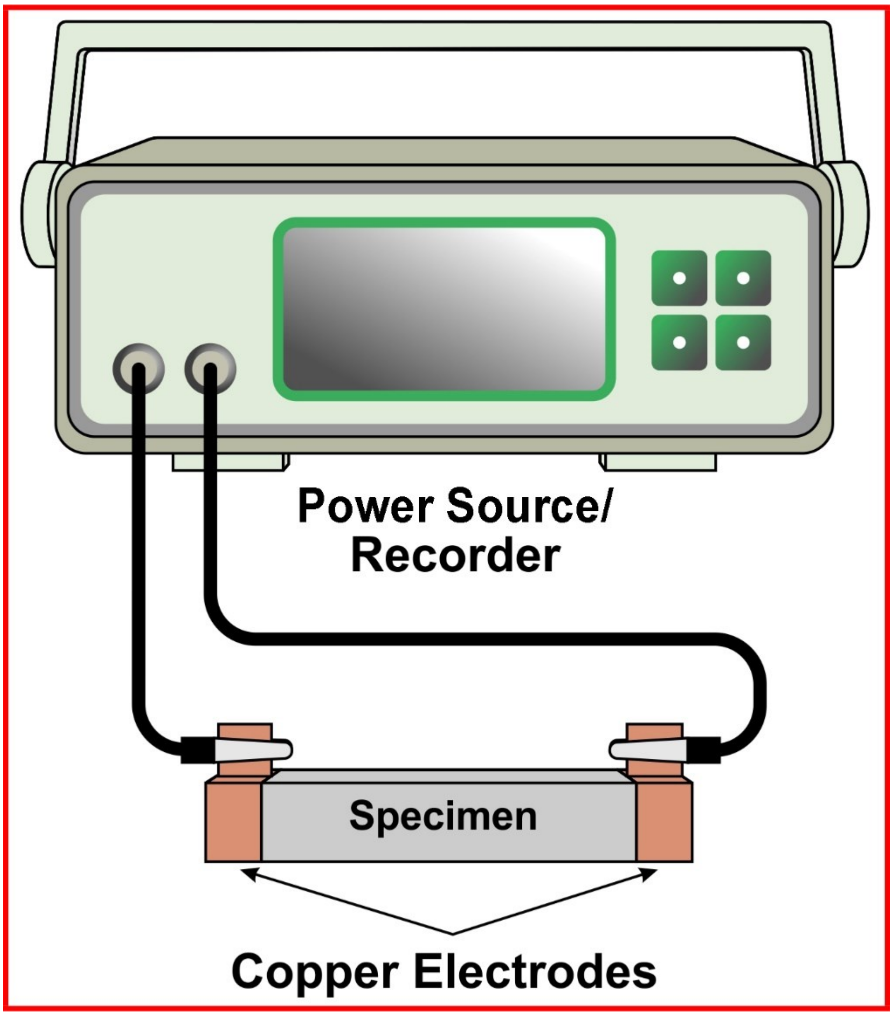

Fig. S3. Schematic image from the system used for resistivity measurement.

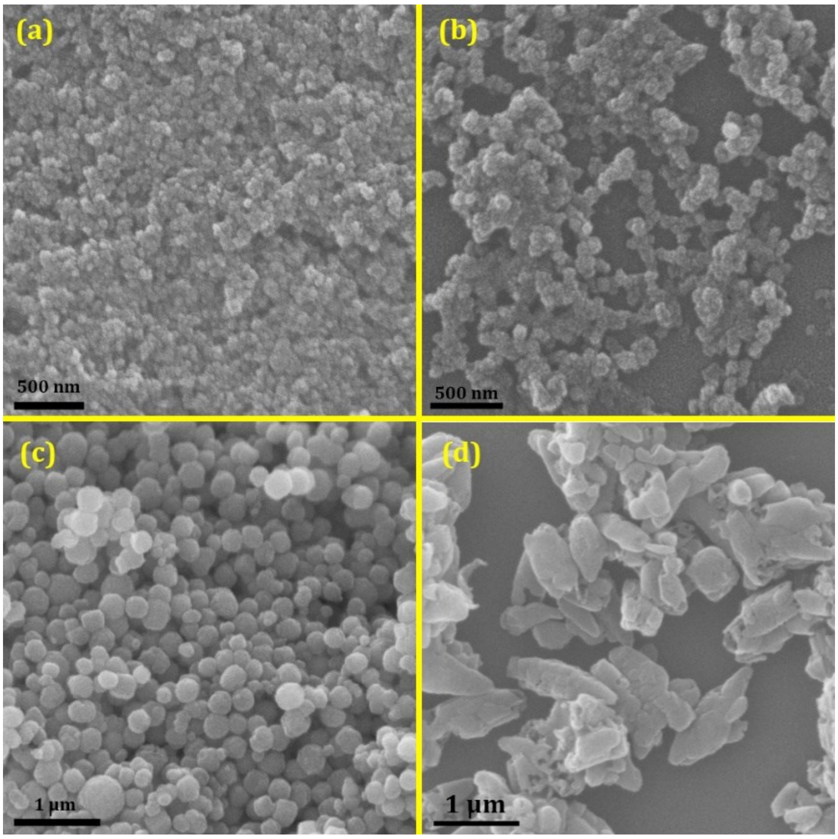

Fig. S4. SEM micrographs from different magnetite sources.

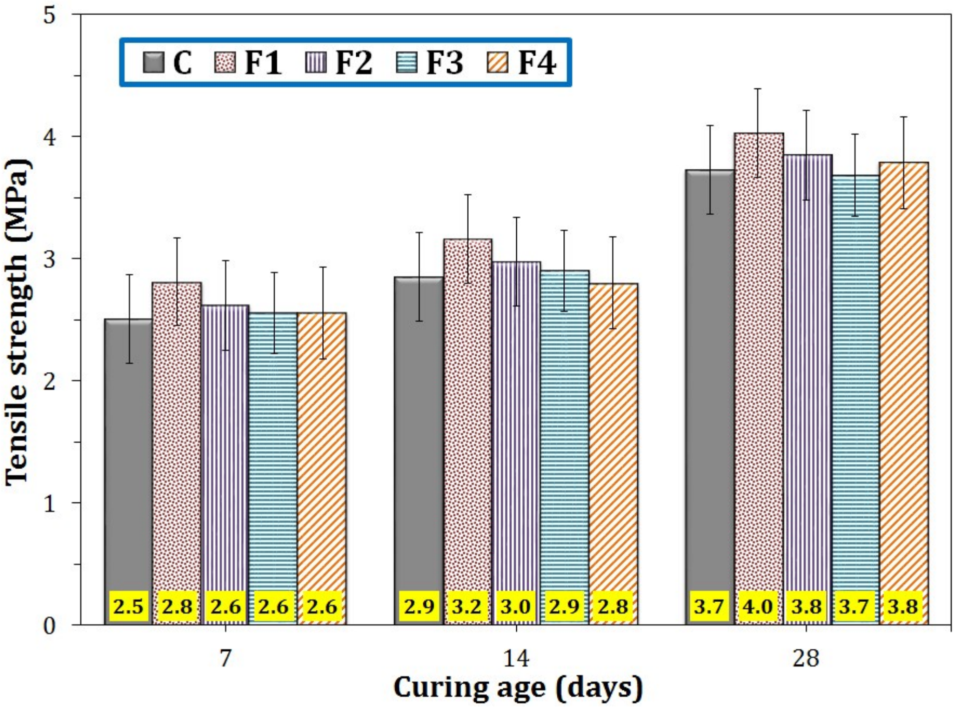

Fig. S5. Tensile strength of the cement paste containing 0.2 wt.% Fe_3O_4 with different particle sizes at different hydration ages.

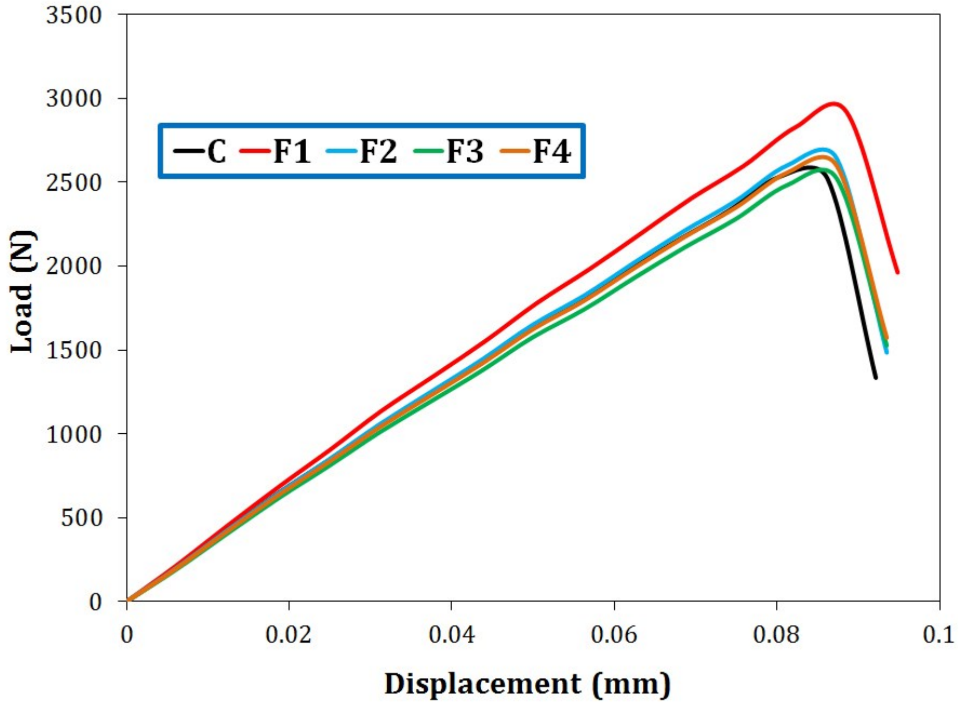

Fig. S6. Tensile load-displacement curves for the cement paste containing 0.2 wt.% Fe_3O_4 with different particle sizes cured for 28 days.

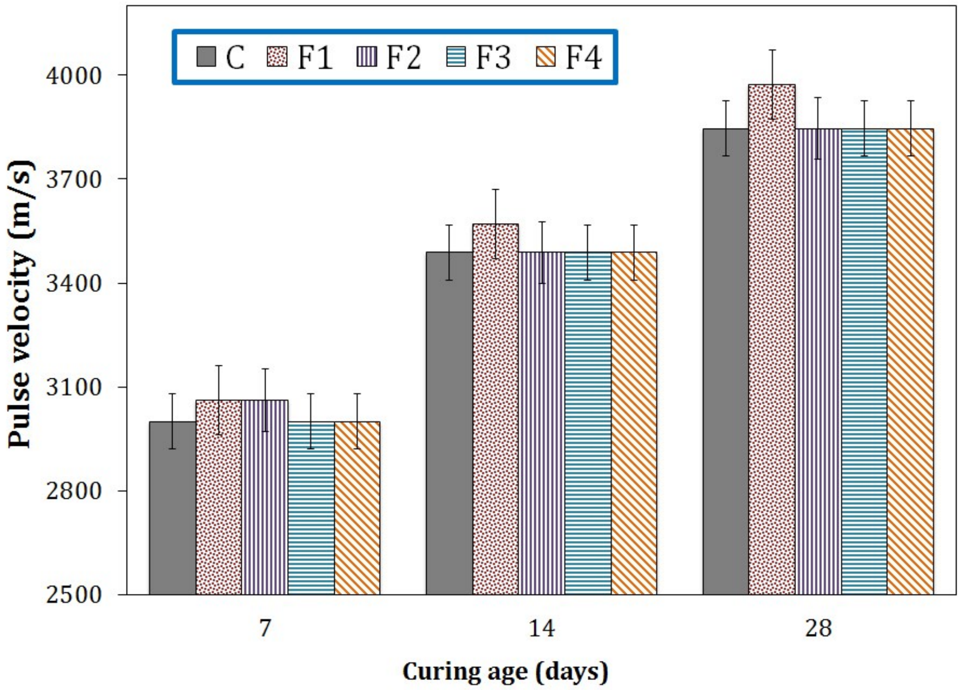

Fig. S7. The velocity of ultrasonic pulses for the cement paste containing 0.2 wt.% Fe_3O_4 with different particle sizes at 7, 14 and 28 days of hydration.

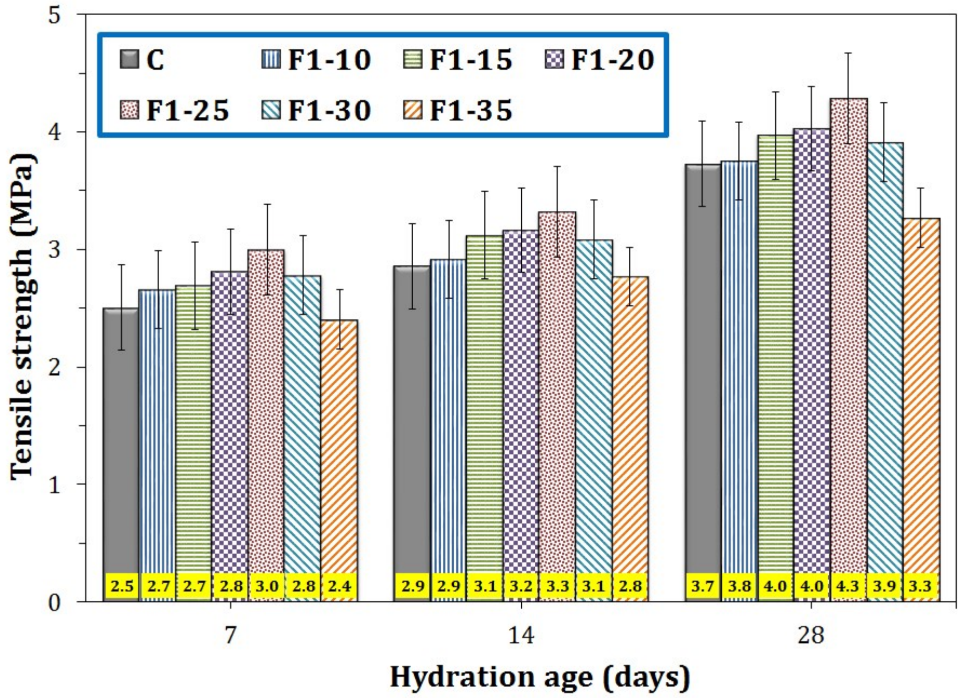

Fig. S8. The tensile strengths of the cementitious samples containing different amounts of F1 nano-Fe_3O_4 at 7, 14 and 28 days of hydration.

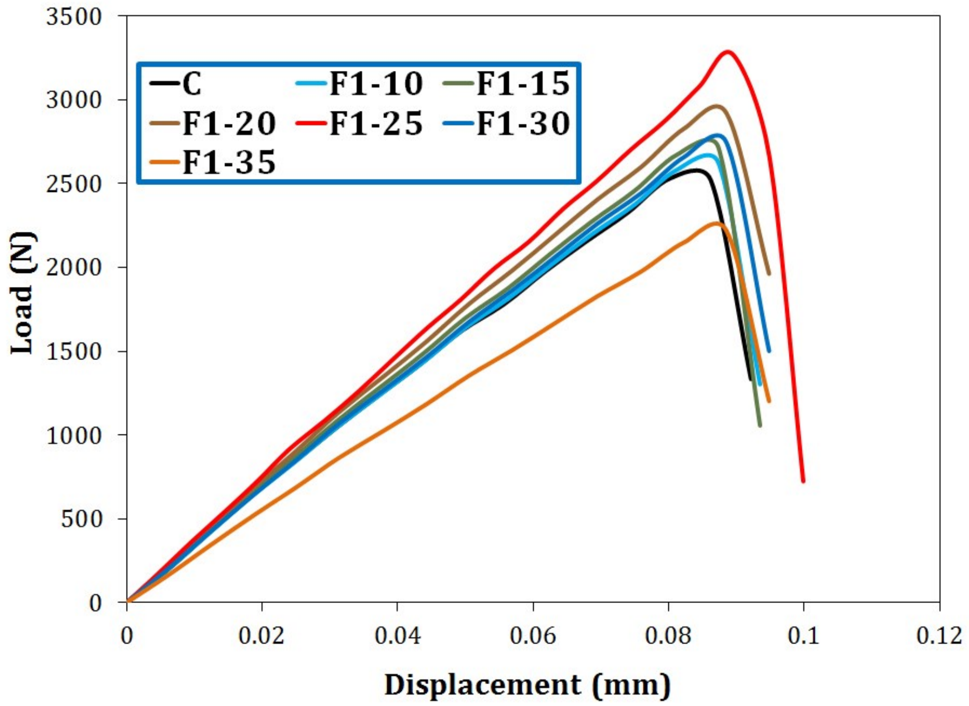

Fig. S9. Tensile load-displacement curves of the cement paste containing different amount of F1 nano-Fe_3O_4 cured for 28 days.

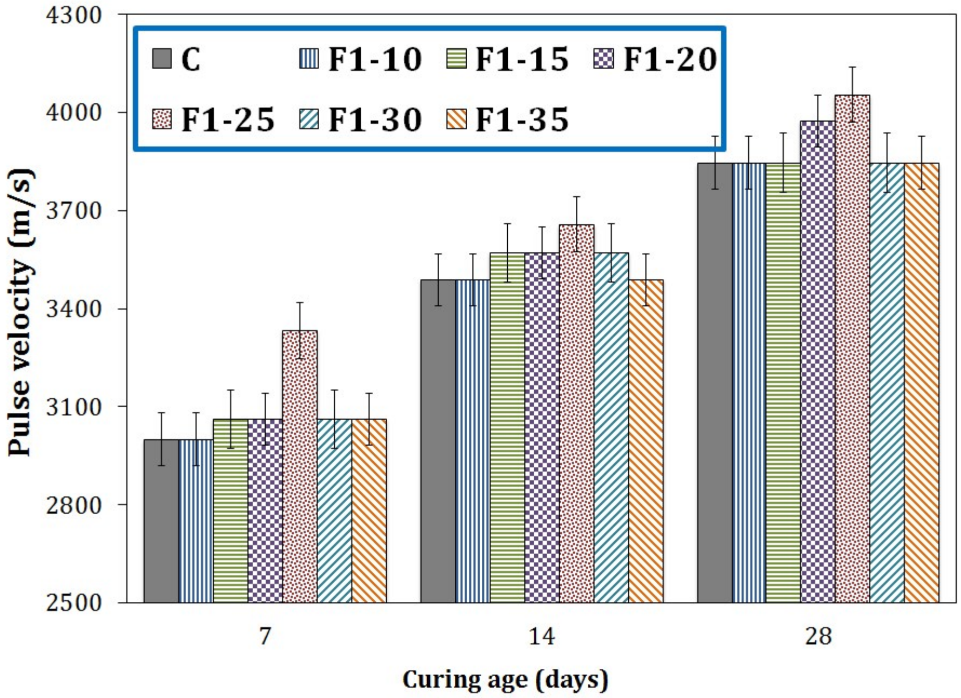

Fig. S10. The velocity of ultrasonic pulses for the cement paste containing different amount of F1 nano-Fe_3O_4 at 7, 14 and 28 days of hydration.
